# Study on the Micro-Mechanism of Corrosion Deterioration of Concrete Under Sulfate Attack Environment

**DOI:** 10.3390/ma18122904

**Published:** 2025-06-19

**Authors:** Yuzhou Sun, Mengjie You, Xiaosan Yin, Dongchang Hou, Jimin Li, Xiangming Zhou

**Affiliations:** 1 School of Civil and Transportation Engineering, Henan University of Urban Construction, Pingdingshan 467036, China; sunyz@zut.edu.cn; 2 Henan Mechanics and Engineering Structures Engineering Research Center, Zhengzhou 451197, China; yinxiaosan_3@126.com; 3 School of Architecture and Engineering, Zhongyuan University of Technology, Zhengzhou 451197, China; 15093638665@163.com (M.Y.); li3135752022@163.com (J.L.); 4 Department of Civil and Environmental Engineering, Brunel University of London, London UB8 3PH, UK; xiangming.zhou@brunel.ac.uk

**Keywords:** concrete, Na_2_SO_4_ erosion, compressive strength, corrosion resistance factor, nanoindentation, K-means cluster analysis

## Abstract

To investigate the influence of the water–cement ratio and erosion patterns on the deterioration of concrete in a sulfate corrosion environment, concrete specimens with different water–cement ratios were immersed in Na_2_SO_4_ solutions of varying concentrations (0%, 5%, and 8%). The immersion times were set at 0 days, 30 days, 60 days, and 90 days. Macro-scale compressive strength tests and micro-scale performance tests were conducted to obtain the damage morphology, micro-scale elastic modulus, and hardness of eroded concrete. Additionally, K-means clustering analysis was used to analyze the micro-mineral phases of the specimens, and SEM and XRD were employed to reveal the degradation mechanisms of sulfate erosion on the microstructure of concrete. The results indicated that the erosion products of calcium aluminate and gypsum in concrete gradually increased with the increase in Na_2_SO_4_ solution concentration and immersion time. In the early stages of erosion, the compressive strength and corrosion resistance coefficient of concrete showed a temporary upward trend, which then decreased as the erosion depth increased. From a microstructural perspective, erosion had a significant impact on the internal structure of concrete, while the elastic modulus and hardness of hydrated calcium silicate and calcium hydroxide under erosion showed relatively minor changes, both exhibiting a gradual decrease. The volume fraction of microporous pores gradually increased, further exacerbating the depth and extent of erosion.

## 1. Introduction

In the acidic and alkaline soil and water environments, the infiltration of sulfate will trigger a series of complex chemical reaction chains within the concrete, which will then lead to volume expansion and cracking phenomenon, laying potential safety hazards for the concrete structure, posing a serious threat to its long-term durability and overall safety [1]. When concrete is subjected to dry and wet cycling, its deterioration process shows the development trend from the surface to the interior, regardless of the type of exposure to salt lake brine [2]. Under the dual action of dry and wet cycling and sulfate attack, the expansion of concrete is lower than that of cement paste, and increases significantly with an increasing concentration of sulfate solution [3]. From the microscopic point of view, the corrosion products formed by concrete in chloride–sulfate mixed solution showed a dense cluster-like morphology and were characterized by a high elastic modulus [4]. In the Na_2_SO_4_ erosion environment, the admixture of basalt fibers effectively improves the damage pattern of concrete and increases its compressive strength, but too high an admixture of basalt fibers will instead lead to a decrease in mechanical properties [5].

In a Na_2_SO_4_ erosion environment, the incorporation of gypsum slag cement was able to increase the compressive strength and the corrosion resistance coefficient of concrete at the beginning of the curing age; however, with the increase of curing age, the concrete exhibited a strength softening type of damage pattern [6]. Under long-term immersion erosion conditions, concrete specimens with smaller dimensions and higher water–cement ratios exhibit poorer erosion resistance. Furthermore, the degree of erosion caused by long-term complete immersion is more severe than that caused by wet–dry cycles. Here, “small” refers to specimens with smaller volumes and relatively larger surface areas, which results in greater exposure to the erosive medium and exacerbates the corrosion effect [7]. In 5% concentration Na_2_SO_4_ solution, the rate of decline of durability index of concrete decreases gradually with the increase of strength grade, and has a similar pattern in clear water, but the rate of decline is relatively slower [8]. For recycled plastic concrete, the compressive strength of recycled plastic concrete in 3%, 5%, and 7% concentrations of Na_2_SO_4_ solution increased during the first 180 d, after which it began to decline and was lower than the compressive strength of ordinary concrete [9]. In conclusion, in acidic and alkaline soil and water environments, Na_2_SO_4_ reacts with cement stones and aggregates in concrete to produce products such as gypsum and calomel, which have lower hardness, and at the same time leads to the loss of calcium ions from the concrete, which destroys the surface protective layer and strength of the concrete, thus accelerating its erosion and aging process [10,11,12,13,14].

With its high load and displacement resolution, the nanoindentation instrument is widely used to test the micro- and nanoscale mechanical properties of material surfaces, including hardness, elastic modulus, plastic strain, film interfacial bond strength, and material fatigue properties [15]. The work of Feng Yin Du et al. [4]. revealed the micromechanical response of reinforced concrete under corrosion of different solutions, in particular, the chloride–sulfate mixed solution. The corrosion products formed in the chloride–sulfate mixture show a unique dense cluster morphology with a high elastic modulus, which further proves the value of nanoindentation technique in corrosion studies.

This progressive process can have significant adverse effects on the stability and safety of structures in long-term use, especially for reservoir concrete exposed to underwater environments for long periods of time. The current trend of research on the corrosion deterioration of concrete under a sulfate erosion environment is still focused on the macro performance test and a lack of in-depth analysis of its micro-mechanisms; therefore, this study utilizes nanoindentation technology to test the modulus of elasticity and hardness of concrete under long-term immersion in Na_2_SO_4_ solution, and K-means clustering to analyze the micro-mineral composition of the cement mortar, to reveal the micro-mechanisms of the corrosion deterioration of concrete, in order to provide scientific guidance for practical engineering, a mechanism of concrete corrosion deterioration, in order to provide scientific guidance for practical engineering.

## 2. Test and Analysis Methods

### 2.1. Raw Materials

Cement was selected as ordinary silicate cement with a strength grade of 42.5; physical properties are shown in Table 1 and chemical composition is shown in Table 2. Fine aggregate was selected as well-graded natural river sand; coarse aggregate was selected as continuously graded crushed stone, of which 35% was crushed stone with a particle size of 5~10 mm and 65% was crushed stone with a particle size of 10~20 mm; mixing water was adopted as laboratory tap water; mineral admixture was adopted as Class I fly ash, the basic properties are shown in Table 3; soaking solution was prepared by anhydrous Na_2_SO_4_, with diluted concentration of 5% and 8% (commonly used for accelerated testing), respectively. The basic properties are shown in Table 3; the soaking solution was prepared by anhydrous Na_2_SO_4_, and the diluted concentrations were 5% and 8%, respectively.

### 2.2. Specimen Production

The size of the concrete specimen is 150 mm × 150 mm × 150 mm, and the fit is shown in Table 4. According to the “Standard for Test Methods of Physical and Mechanical Properties of Concrete” (GB/T50081-2019) [16], concrete was poured, and the specimens were molded and left to stand for 1 d~2 d in a room with a temperature of 20 °C ± 5 °C and a relative humidity of more than 50%, and then numbered and labeled, unmolded, and placed in a curing room for 28 d. Then, it was immersed in a Na_2_SO_4_ solution with a concentration of 0%, 5%, and 8%, with the design. The immersion cycles were 0, 30, 60, and 90 d. When the immersion time of the test blocks reached the immersion cycle, their compressive strength, modulus of elasticity, and hardness were tested.

### 2.3. Compressive Strength and Corrosion Resistance

Test the compressive strength of concrete according to the Standard for Test Methods of Physical and Mechanical Properties of Concrete (GB/T50081-2019), calculated according to the following formula:

(1) $$f_{\mathrm{cc}}=F/A$$

where concrete cube specimen compressive strength (MPa); calculation results should be accurate to 0.1 MPa; *F* for the specimen destructive load (N); *A* for the specimen pressure area (mm^2^).

According to the Standard for Long-term Performance and Durability Test Methods for Ordinary Concrete (GB/T50082-2024) [17], the corrosion resistance coefficient is tested after the design immersion cycle and is calculated according to the following formula:

(2) $$K_{f}=\frac{f_{\mathrm{cn}}}{f_{c0}}\times 100\%$$

where for the compressive strength of the specimen after erosion nd corrosion resistance coefficient (%); for the compressive strength of the specimen after erosion nd (MPa); and subjected to sulfate corrosion specimen with the same age of the standard curing of a group of comparative concrete specimens of the compressive strength of the measured value (MPa), accurate to 0.1 MPa.

### 2.4. Changes in the Apparent Morphology of the Test Specimens

Changes in the appearance of test specimens are intuitive and direct test indicators. By observing changes in the concrete surface mortar, cracks, etc., the deterioration process can be clearly reflected. Changes in the concrete surface after sulfate erosion vary depending on environmental conditions. Therefore, changes in appearance can effectively reflect the deterioration process of concrete under the action of sulfate erosion.

### 2.5. SEM Test

After conducting cube compressive strength tests on specimens corresponding to the respective erosion age periods, samples were taken from the edges and center of the specimens. The crushed concrete specimens were immersed in anhydrous ethanol for 2 days, then dried in a 45 °C drying oven for 3 days. Prior to scanning electron microscopy (SEM), all test specimens were subjected to gold sputtering. Record their microstructural morphology and compare it with the microstructural morphology of specimens with an erosion age of 0 days.

To further investigate the mechanism of C-S-H erosion under the influence of Na_2_SO_4_, microscopic observations (SEM) were conducted on samples with different corrosion durations. Samples were selected as follows: untreated (GS1-0), immersed in an 8% Na_2_SO_4_ solution for 30 days (GS1-8-30), immersed in an 8% Na_2_SO_4_ solution for 60 days (GS1-8-60), soaked in water for 90 days (GS1-W-90), soaked in a 5% Na_2_SO_4_ solution for 90 days (GS1-5-90), and soaked in an 8% Na_2_SO_4_ solution for 90 days (GS1-8-90) subjected to scanning electron microscopy (SEM) analysis.

### 2.6. XRD Test

Remove the sample soaked in anhydrous ethanol, allow it to dry, and grind it into a powder using an agate mortar until there are no visible particles. Then, sieve the ground concrete powder using a sieve with a mesh size of 0.075 mm. Finally, place the sieved concrete on a glass slide. Use an X-ray diffractometer to analyze the chemical composition of the sample.

To further investigate the mechanism of C-S-H erosion under the influence of Na_2_SO_4_, microscopic observations (XRD) were conducted on samples with different corrosion durations. Samples were selected as follows: untreated (GS1-0), immersed in an 8% Na_2_SO_4_ solution for 30 days (GS1-8-30), immersed in an 8% Na_2_SO_4_ solution for 60 days (GS1-8-60), soaked in water for 90 days (GS1-W-90), soaked in a 5% Na_2_SO_4_ solution for 90 days (GS1-5-90), and soaked in an 8% Na_2_SO_4_ solution for 90 days (GS1-8-90) subjected to XRD phase analysis.

### 2.7. Nanoindentation Test

#### 2.7.1. Test Principle

Nanoindentation is an advanced technique to measure the mechanical properties of materials at the nanoscale [18], using hardness and elastic modulus to characterize the mechanical behavior at small scales [19]. Based on the classical theory Oliver–Pharr method, the F-h curve is measured and the modulus and indentation hardness are calculated:

(3) $$E_{r}=\frac{\sqrt{\pi }}{2\beta \sqrt{A_{c}}}S$$

(4) $$H=\frac{F}{A_{c}}$$

(5) $$S=\frac{dF}{dH}|h_{\max}$$

where *β* is a dimensionless correction factor related to the shape of the indenter, *β* = 1.05 when a Berkovich indenter is used; *S* is the indentation unloading stiffness; and *A_c_* is the contact area at the maximum depth of indentation, *h_max_*, which is a function of the contact depth, *h_c_*, at *h_max_* [20]. For isotropic homogeneous materials, the modulus of elasticity is calculated according to the following equation:

(6) $$\frac{1}{E_{r}}=\frac{\left(1-v^{2}\right)}{E}+\frac{\left(1-v_{i}^{2}\right)}{E_{i}}$$

where *E* and *v* are the modulus of elasticity and Poisson’s ratio of the material under test, and *E_i_* and *v_i_* are the modulus of elasticity and Poisson’s ratio of the indenter, respectively.

#### 2.7.2. Test Methods

The concrete specimens cured for 28 d were first soaked in anhydrous ethanol for 48 h to stop the cement hydration reaction and to ensure that the microstructure of the specimens would no longer change in the subsequent processing. Then, they were placed in a drying oven at 45 °C for 48 h in order to remove the moisture in the specimens for subsequent cutting and grinding treatment. The specimens were cut into small cubic pieces of 15 mm × 15 mm × 15 mm for subsequent grinding and polishing treatment. Place the cut specimen in a cold-set mold and pour curing agent into the mold, the thickness of which should be about 2/3 of the height of the cold-set mold, in order to fix the specimen so that it will not move or be deformed in the subsequent grinding and polishing process.

After the curing is completed, the samples are taken out and placed on the grinding and polishing machine and ground using different sizes of sandpaper. The grinding time was not less than 20 min per grit of sandpaper to ensure that the surface of the specimen gradually became flat and smooth. During the grinding process, anhydrous ethanol needs to be continuously sprayed on the grinder to prevent overheating of the specimen and the generation of excessive dust. After the grinding was completed, the flannel cloth was replaced and the polishing process was continued with 3 μm, 1 μm, and 0.3 μm diamond suspensions for 2 h in order to further remove the tiny scratches and unevenness on the surface of the specimens to achieve a mirror effect on the surface, which was easy to observe and analyze under the microscope.

The polished specimen was placed in an ultrasonic cleaner for 10 min, in order to remove the residual powder and impurities on the surface of the specimen and to ensure the cleanliness of the specimen surface for subsequent microstructure observation or analysis.

**Figure 1:**
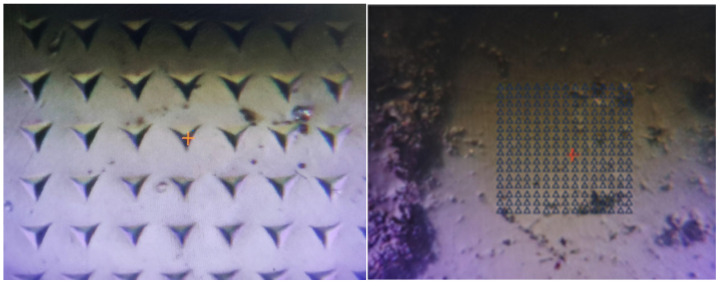
Schematic diagram of mortar indentation array.

An Agilent Nano Indenter G200 nanoindenter (Santa Clara, CA, USA) was used to test the micromechanical properties of the specimens. The maximum indentation depth was set at 2000 nm and the indentation spacing was 20 μm. Then, 15 × 15 dot matrix indentation was performed for each group, as shown in Figure 1. The loading process was loaded for 10 s, held for 5 s, unloaded for 10 s. The thermal drift index was controlled to be 0.04 nm/s during the test.

#### 2.7.3. Cluster Analysis

Most of the data analysis of nanoindentation is performed by inverse convolution analysis, but it suffers from problems such as high computational effort and overfitting [21]. In the field of machine learning, cluster analysis is an unsupervised learning algorithm whose main purpose is to automatically categorize similar samples into the same class. Commonly used similarity calculation methods include Euclidean distance method, Manhattan distance method, and Chebyshev distance method, etc., among which K-means and K-medoids are common algorithms based on Euclidean distance method.

In this paper, the K-means algorithm [22] is selected, which calculates the distance of sample points from the center of mass of class clusters and classifies the sample points with closer distances into clusters of the same class. The principle of K-means clustering calculation is to select K initial centers of mass, and then calculate their Euclidean distances from each center of mass for each of the remaining sample points, and classify them into clusters with the closest centers of mass. Next, the center of mass of each new cluster is calculated and all sample points are reclassified. After all sample points have been reclassified, the center-of-mass position of each cluster is recalculated based on the new division and the distance from each sample point to the center-of-mass of each cluster is calculated iteratively until convergence. Set E as the sum of the squares of the distances of the points within all data groups from their respective centroids [23], as shown in the following equation:

(7) $$E={\sum_{i=1}^{k}\sum_{x\in C_{i}}dist\left(x_{i},x_{j}\right)}^{2}$$

where denotes the Euclidean distance between a sample point having *p* dimensions and i.e.,

(8) $$dist(x_{i},x_{j})=\sqrt{\sum_{a=1}^{p}{\left(x_{i}^{a}-x_{j}^{a}\right)}^{2}}$$

When E reaches the minimum, k clusters C1, C2, … Ck and their clustering centers O1, O2, … Ok are obtained.

**Figure 2:**
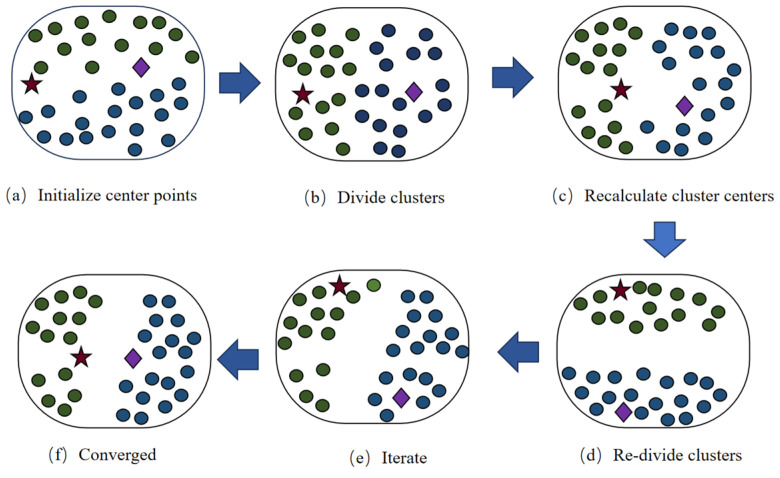
Schematic diagram of K-means clustering process.

The K-means clustering process is schematically shown in Figure 2.

## 3. Analysis of Test Results

### 3.1. Strength and Corrosion Resistance Coefficient Deterioration Pattern with Erosion Age

**Figure 3:**
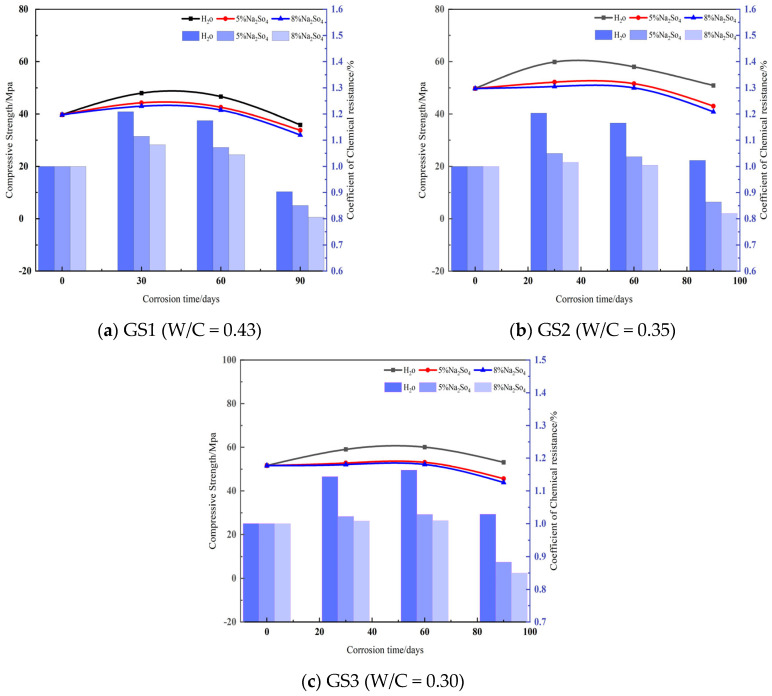
Compressive strength and corrosion resistance coefficient of concrete under different erosion conditions.

**Figure 4:**
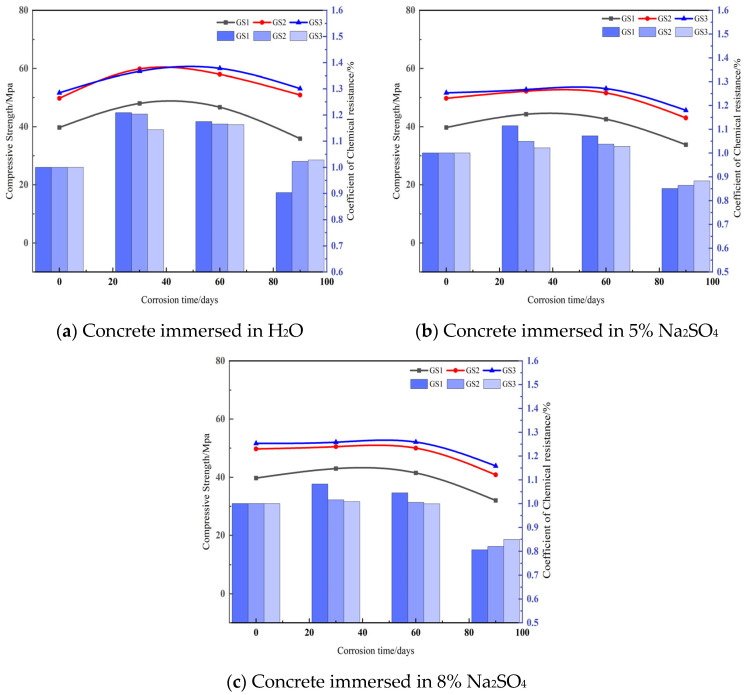
Compressive strength and corrosion resistance of concrete at different concentrations.

Figure 3 and Figure 4 show the compressive strength and corrosion resistance coefficients of the specimens at different water–cement ratios and at different concentrations, respectively. With the extension of age, it can be seen that the compressive strength increased in the early stage, but after that, all of them showed a significant decrease. This phenomenon can be explained by the fact that in the early stage of concrete erosion, sulfate ions have not yet penetrated into the internal pores of concrete in large quantities. At this time, the hydration products (e.g., calcite, gypsum, etc.) generated by the internal hydration reaction of the concrete filled the pores of the hardened paste and increased the densification of the concrete. This filling effect resulted in the phenomenon of increasing rather than decreasing the strength of the specimens [24]. In the later stages of erosion, the internal porosity gradually decreases as the specimen densities continue to increase. This leads to limited space within the concrete, which in turn leads to the accumulation of more harmful products. The limited space triggers the expansion products to generate tensile stresses that gradually increase [25]. The compressive strength and erosion coefficient of concrete show a decreasing trend with increasing sulfuric acid concentration and soaking time. This is due to the fact that the accumulation of harmful substances leads to tensile stresses exceeding the tensile strength of concrete, which triggers the formation of cracks in the specimens. These cracks become channels for harmful ions, accelerating their invasion and the generation of harmful products, forming a vicious cycle that ultimately leads to the destruction of the specimen.

Analyzing Figure 3, it can be seen that the trends of compressive strength and the erosion coefficient of concrete are basically the same under different water–cement ratios, whether in clear water or different concentrations of Na_2_SO_4_ solution. In GS1, the maximum compressive strength and erosion coefficient of the fresh-water group and 5% and 8% Na_2_SO_4_ were reached at 30 d. The peak compressive strengths were 47.98 MPa, 44.26 MPa, 42.98 MPa, and the peak erosion coefficients were 1.21, 1.11, and 1.0, respectively. In GS2, the fresh-water group and 5% and 8% Na_2_SO_4_ reached the maximum compressive strength and erosion coefficient at 30 d The peak compressive strengths were 59.85 MPa, 52.19 MPa, 50.51 MPa, and the peak erosion coefficients were 1.20, 1.05, and 1.02, respectively; in GS3, the fresh-water group and 5% and 8% Na_2_SO_4_ reached the maximum at 60 d. The peak compressive strengths were 60.05 MPa, 53.07 MPa, 52.10 MPa, and the peak erosion coefficients were 1.16, 1.03, and 1.03, respectively. For compressive strength and erosion coefficient, the peak compressive strength was 60.05 MPa, 53.07 MPa, 52.10 MPa, and the peak erosion coefficient was 1.16, 1.03, 1.01, respectively.

With the increase of sulfuric acid concentration, both compressive strength and erosion coefficient show a decreasing trend. The reason for this phenomenon is mainly due to the formation of hydration products inside the concrete and the erosion effect of sulfate ions. At the initial stage, the calcium alumina and gypsum generated by the hydration reaction filled the pores and improved the densification of the concrete, leading to an increase in compressive strength. However, with the prolongation of time and the increase of sulfuric acid concentration, the porosity gradually decreases, and the internal space becomes restricted, which leads to the accumulation of harmful products and the generation of tensile stresses, and ultimately triggers the cracks, forming a vicious cycle, resulting in the decline of compressive strength and corrosion resistance.

By analyzing Figure 4, it can be seen that different water–cement ratio concrete in different concentrations of corrosion after the change rule of compressive strength is basically the same, mainly the first rise after the decline of two stages. For concrete immersed in water, for specimens GS1, GS2, GS3 the compressive strength peak ages were 30 d, 30 d, 60 d, corrosion resistance coefficient peak age was 30 d, the peak compressive strengths were 47.98 MPa, 59.85 Mpa, 60.05 Mpa, and the corrosion resistance coefficients of the peak were 1.21, 1.20, 1.16. The peak compressive strengths of concrete soaked in 5% Na_2_SO_4_ were 44.26 Mpa, 52.19 Mpa, 53.07 Mpa, and the peak corrosion resistance coefficients were 1.11, 1.05, 1.02, respectively; the peak compressive strengths of concrete soaked in 8% Na_2_SO_4_ were 42.98 Mpa, 50.51 Mpa, 52.10 Mpa, and the peak corrosion resistance coefficients were 1.08, 1.02, 1.01, respectively, showing that the specimens with 0.43 water–cement ratio reached the maximum peak value, and with the decrease of water–cement ratio, the peak corrosion resistance coefficient reached the maximum value. The peak values of the corrosion resistance coefficients were 1.08, 1.02, and 1.01, respectively, which showed that the specimens with 0.43 water–cement ratio had the largest peak corrosion resistance coefficients, and with the decrease of water–cement ratio, the age of concrete specimens with peak compressive strengths was delayed and the growth of the compressive corrosion resistance coefficients was reduced.

The main reason for this phenomenon Is the effect of water–cement ratio on the microstructure and properties of concrete. A higher water–cement ratio helps in the formation of hydration products and enhances the densification of the concrete, thereby increasing the compressive strength and corrosion resistance coefficient. The strength of concrete rises to a peak when initially immersed in fresh water and then begins to decrease due to the erosion of sulfate ions. In 5% and 8% Na_2_SO_4_ immersion, the peak compressive strength and corrosion resistance coefficient decreased gradually with the increase of sulfuric acid concentration, reflecting the weakened durability of concrete in high concentration corrosive environments. In addition, a decrease in the water–cement ratio resulted in a delay in the peak compressive strength, indicating that lower water–cement ratios reduced the initial strength response of the concrete.

### 3.2. Appearance Changes in Specimens

Under natural immersion conditions in water, concrete specimens with different water–cement ratios exhibit distinct changes in appearance as erosion time increases. The specific trends and relationships are outlined in Table 5, illustrating the correlation between erosion time and changes in specimen appearance. As shown in Table 5, since the test was conducted solely under water immersion, the surface changes in the concrete specimens were extremely subtle and barely noticeable. As erosion time increases, some pores appear on the surface of the concrete specimens, but no cracks have yet formed.

In concrete tests naturally immersed in a 5% Na_2_SO_4_ solution, as erosion time increases, the surface changes of concrete specimens with different water–cement ratios exhibit a distinct trend. These changes continue to intensify over time.

As shown in Table 5, during the first 60 days of erosion, compared to the 30-day mark, a layer of white material covered the surface of the specimens, which may be AFt and gypsum. At the same time, the concrete surface developed a small number of microcracks, and the number of pores gradually increased. After 90 days of immersion erosion, the specimen surfaces remained covered with white substances, and the edges of the specimens began to exhibit small, discontinuous notches and damage. The pores on the surface not only enlarged but also increased sharply in number.

In immersion tests in an 8% Na_2_SO_4_ solution, the appearance changes of concrete specimens also exhibited a clear trend as erosion time increased.

As shown in Table 5, after 30 days of immersion, the concrete surface exhibited an increasing number of pores, became uneven, and showed signs of sand particle detachment. After 60 days of immersion erosion, the specimen surface was covered with a layer of white material, damage at the edges worsened, and the distribution of cracks gradually increased. After 90 days of immersion erosion, the test specimens became brittle and could be easily broken by hand, with more pronounced sand particle detachment, severe damage, and obvious signs of surface destruction, and the specimens lost their original cohesion.

Compared to GS1 and GS3, due to the higher water–cement ratio of GS1, the increase in pores was more severe and denser, resulting in a more fragile surface and more pronounced deterioration.

### 3.3. Analysis of SEM Test Results

The destructive effects of sulfate solutions on concrete can be analyzed from both physical and chemical perspectives. Physical damage refers to the formation of expansive crystals after the solution penetrates the concrete, triggering and exacerbating crack formation; chemical damage involves reactions between the solution and the hydration products of cement within the concrete, leading to the formation of expansive substances such as AFt and ${\mathrm{CaSO}}_{4}\cdot {2H}_{2}O$ at crack sites. In Na_2_SO_4_ solutions, unhydrated cement particles react with components in the solution to form new hydration products, which expand within cracks. As time progresses and Na_2_SO_4_ concentration increases, the size and quantity of these products also increase. The newly formed hydration products accumulate and expand within cracks, aiding in the self-repair of concrete. However, when Na_2_SO_4_ concentration is excessively high, the hydration products expand excessively, reducing concrete bond strength and generating crystallization pressure, which may lead to secondary cracking [9].

**Figure 5:**
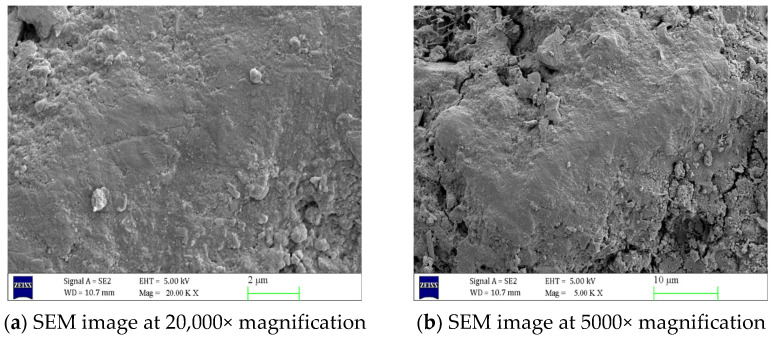
Scanning electron microscope images of concrete before erosion at different magnifications.

**Figure 6:**
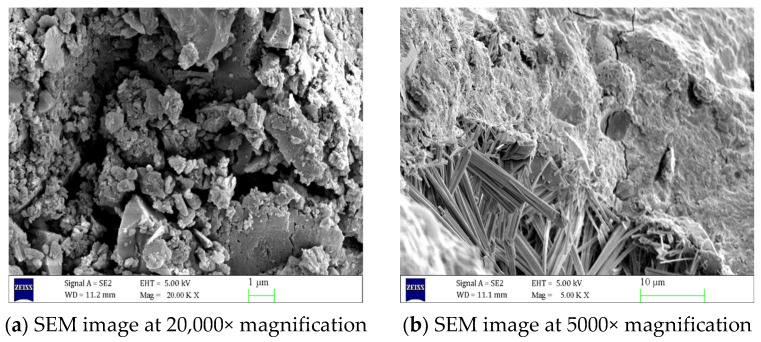
Scanning electron microscope images of concrete eroded by 5% Na_2_SO_4_ for 90 days at different magnifications.

**Figure 7:**
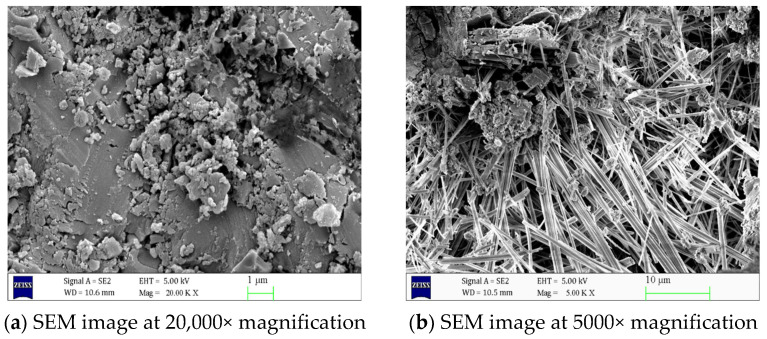
Scanning electron microscope images of concrete after 90 days of 8% Na_2_SO_4_ erosion at different magnifications.

As shown in Figure 5, in uncorroded concrete, C-S-H gel and AFt are uniformly distributed and fill the interior of the concrete, resulting in a compact and orderly internal structure with tightly connected aggregates and nearly invisible cracks. However, as shown in Figure 6, concrete specimens corroded in a 5% Na_2_SO_4_ solution for 90 days exhibit significant changes in structural morphology. A large number of needle-like or rod-like AFt crystals have formed at the crack cross-sections, and the volume of AFt has significantly increased. Figure 7 shows that as the concentration of the Na_2_SO_4_ solution increases, Ca(OH)_2_ crystals gradually disappear, C-S-H gel becomes loose, and the number of cracks increases. The volume and quantity of AFt also further increase, with a large number of needle-like or rod-like AFt intertwining and overlapping, filling the interior of the cracks.

### 3.4. Analysis of XRD Test Results

**Figure 8:**
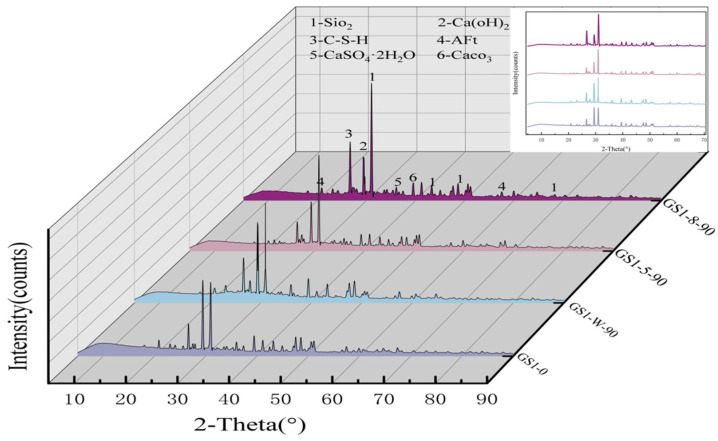
XRD spectra of concrete at different concentrations.

Import the XRD test data into the JADE (6.0) analysis software and use the phase identification function to identify characteristic peaks, as shown in Figure 8. The spectrum shows that the concrete has multiple diffraction peaks, with the main components including silica, gypsum (${\mathrm{CaSO}}_{4}\cdot {2H}_{2}O$), AFt, and C-S-H, among others.

After concrete is exposed to Na_2_SO_4_ corrosion, SO_4_^2−^ reacts with calcium hydroxide (CH) and hydrated calcium aluminate in the cement to form AFt, thereby creating AFt crystals and CaSO_4_·2H_2_O crystals. As shown in Figure 8, uncorroded concrete contains a large amount of Ca(OH)_2_ and C-S-H gel, along with small amounts of initial hydration products of cement, calcium sulfate, and AFt. As corrosion time increases, CH gradually decreases, while the content of calcium carbonate, AFt, and ${\mathrm{CaSO}}_{4}\cdot {2H}_{2}O$ gradually increases. This indicates that in the early stages of corrosion, the specimens are primarily damaged by the physical crystallization of sulfates, while in the later stages, the specimens are simultaneously subjected to both physical crystallization and chemical corrosion damage caused by sulfates. Due to the accumulation of crystallization in the early stages, SO_4_^2−^ is more easily introduced into the concrete, triggering chemical corrosion.

### 3.5. Mineral Image Cluster Distribution

In cementitious materials represented by concrete, the hydration of cement particles with water results in the formation of a viscous cement paste that gradually hardens into a porous solid, leading to significant changes in the microstructure of cement. Concrete is a porous, multiphase, non-homogeneous material, and the hydration products mainly contain hydrated calcium silicate (C-S-H gel), calcium hydroxide (Ca(OH)_2_), and hydrated calcium aluminate (C-A-H), etc. [26].

**Figure 9:**
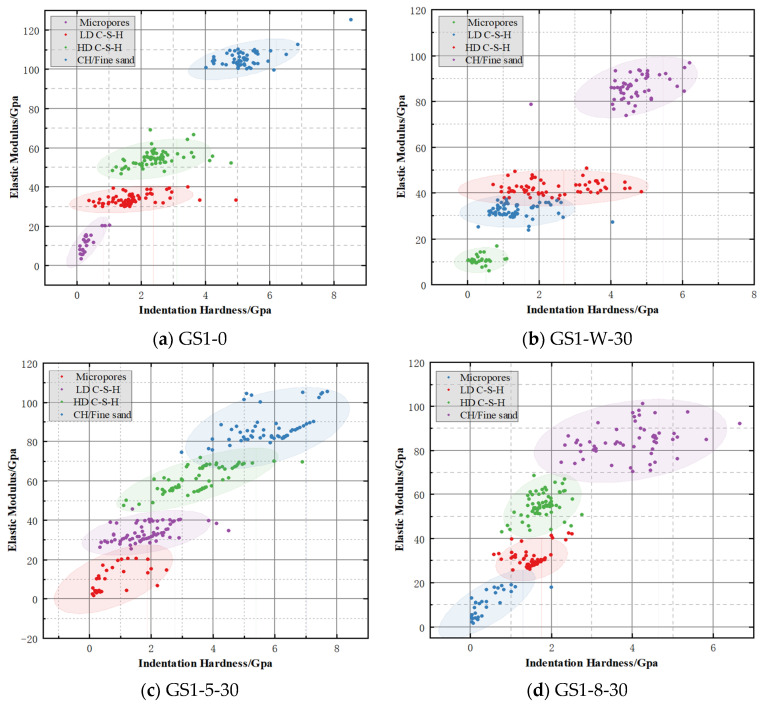

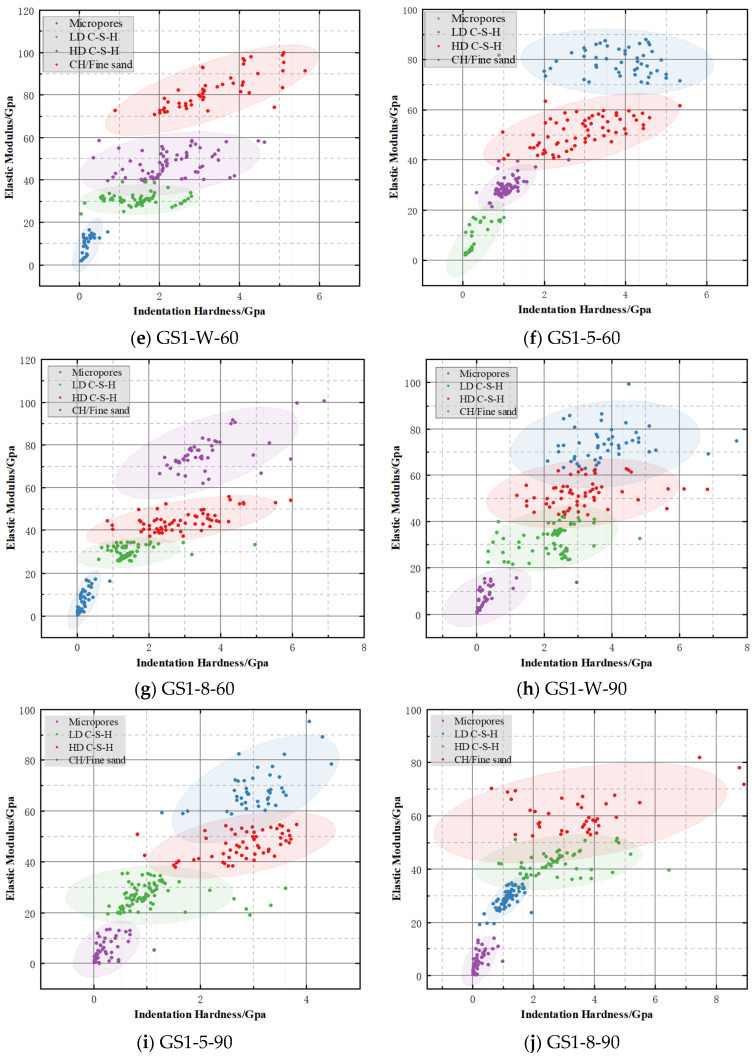
K-means clustering results of concrete specimens under different erosion conditions.

When K-means cluster analysis is used to investigate the modulus of elasticity and hardness at the indentation point of the specimen, the value of K is first determined. The constant K is the final number of clustering categories, which allows a brief classification of the mineral phases of the specimen, which are generally categorized as Micropores, Low-density C-S-H phase (LD C-S-H), High-density C-S-H phase (HD C-S-H), Calcium Hydroxide phase (CH), and Fine sand [27,28]. As shown in Figure 9, Figure 9a–j are without immersion (GS1-0), immersion in water for 30 d (GS1-W-30), immersion in 5% Na_2_SO_4_ for 30 d (GS1-5-30), immersion in 8% Na_2_SO_4_ for 30 d (GS1-8-30), immersion in water for 60 d (GS1-W-60), immersion in 5% Na_2_SO_4_ for 60 d (GS1-5-60), immersion in 8% Na_2_SO_4_ for 60 d (GS1-8-60), immersion in water for 90 d (GS1-W-90), immersion in 5% Na_2_SO_4_ for 90 d (GS1-5-90), and immersion in 8% Na_2_SO_4_ for 90 d (GS1-8-90). It can be seen that the points are mainly distributed in LD C-S-H, HD C-S-H. In the unsoaked concrete, the CH phase represents higher calcium hydroxide, while the low-strength Micropores phase is less, and in the specimens soaked in 8% Na_2_SO_4_ for 90 d, there is a significant decrease in the calcium hydroxide, while the low-strength Micropores phase is higher.

With the increase of concentration and soaking time, CT scans showed changes in the pore structure of concrete, which was manifested by the gradual increase and expansion of colloidal pores or microcracks, and the formation of capillary pores with a diameter of less than 30 μm, and these microscopic changes led to a gradual loosening of the microstructure of concrete, which in turn affected its overall performance [29]. In addition, the increase in micropores also leads to a decrease in the modulus of elasticity of the physical phase measured by an indentation test [30]. The distribution and volume content of the mineral phases under different erosion conditions were analyzed by K-means clustering, as shown in Table 6, with H and E representing the hardness and modulus of elasticity of the material, respectively, and f denoting the percentage of each component. It can be seen that with the increase of concentration and soaking days, the main contents in cement mortar are still LD C-S-H and HD C-S-H. Among them, the content of LD C-S-H in the physical phase increased from 32% in the unsoaked condition to 36.45% in the 90 d soaking in 8% Na_2_SO_4_, while the content of HD C-S-H in the physical phase decreased from 30.22% in the unsoaked condition to 36.45% in the 90 d soaking in 8% Na_2_SO_4_, while the content of HD C-S-H in the physical phase decreased from 30.22% in the unsoaked condition to 36.45% in the 90 d soaking in 8% Na_2_SO_4_. Na_2_SO_4_ for 90 d, while the physical phase HD C-S-H content decreased from 30.22% without soaking to 25.34% at 8% concentration of Na_2_SO_4_.

**Figure 10:**
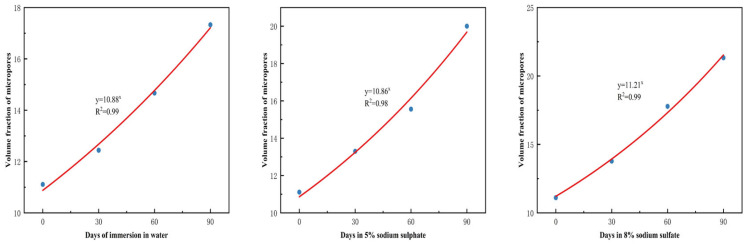
Volume fraction of micropores under different soaking conditions.

The change in the volume fraction of micropore holes was more significant and had an exponential function with the number of days of soaking, as shown in Figure 10. Its content increased from 11.11% when it was not immersed to 21.33% when it was immersed in 8% concentration of Na_2_SO_4_ for 90 d. This shows that the pore space gradually increases under the erosion of Na_2_SO_4_. In addition, the content of CH/Fine sand decreased from 26.67% to 16.89%, which was due to the chemical reaction between Na_2_SO_4_ and calcium hydroxide in the cement, which produced gypsum and other products, resulting in the decrease of calcium hydroxide content. Moreover, the erosion of Na_2_SO_4_ leads to spalling of the concrete surface, enlargement of the gap between cement particles, and exposure of cement particles to the erosive environment, which increases their contact area with Na_2_SO_4_ and exacerbates the loss of calcium hydroxide and unhydrated materials from the cement.

**Figure 11:**
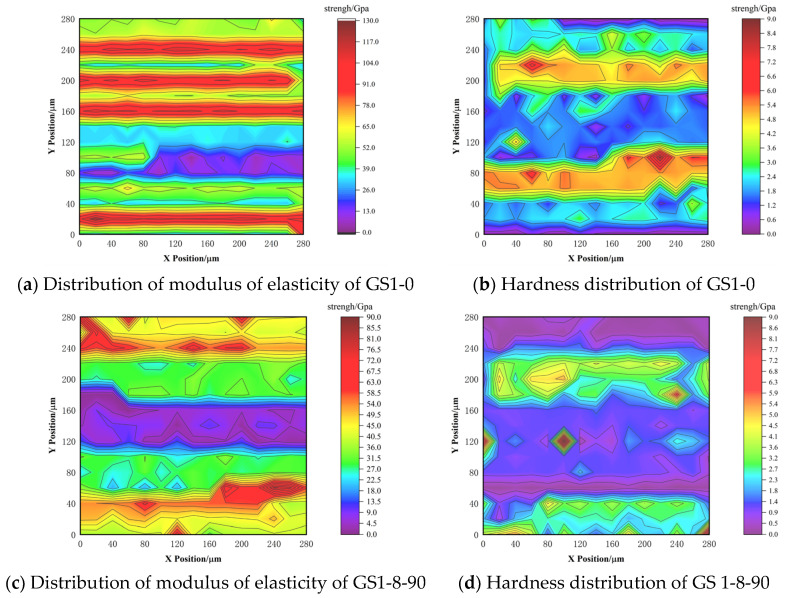
Mineral phase distribution of concrete specimens under different erosion conditions.

As shown in Figure 11, observing the geometrically distributed positions, it can be seen that the content of the Micropores phase increases significantly from GS1-0 to GS1-8-90, while the content of the CH phase decreases dramatically. In both groups GS1-0 and GS1-8-90, the CH and HD C-S-H phases, which have elastic modulus and hardness, are mainly concentrated around the unhydrated cement particles, while the LD C-S-H phase is closely adjacent to the Micropores phase. Meanwhile, the LD C-S-H and HD C-S-H phases were high in content and showed a continuous distribution, which was regarded as the matrix phase of the cement mortar and was crucial in calculating the composite properties. On the contrary, the Micropores phase, the CH phase, and the Fine sand phase have a low content, which are dispersed in the cement mortar and act as intercalation phases [23].

## 4. Conclusions

The effect on compressive strength and corrosion resistance coefficient and the changes in microstructure and micromechanical properties of concrete under erosive conditions have been investigated by experiments and K-means clustering analysis, and the following conclusions can be drawn:

The trends of compressive strength and erosion coefficient of concrete under different water–cement ratios in clear water and different concentrations of Na_2_SO_4_ solutions are basically the same, both showing the law of increasing first and then decreasing. Under the condition of clear water, the compressive strength and erosion resistance coefficient of concrete specimens reached the peak value earlier, and then stabilized due to the absence of erosive influence. In Na_2_SO_4_ solution, with the increase of sulfuric acid concentration, the peak compressive strength and corrosion resistance coefficient of concrete decreased significantly, and the age of reaching the peak was delayed, which was mainly due to the enhanced erosion of sulfate ions on the concrete, resulting in the accumulation of internal harmful products and triggering cracks, which reduced the durability of concrete.

Higher water–cement ratios help to improve the initial strength and corrosion resistance of concrete, but lowering the water–cement ratio delays the onset of peak compressive strength and reduces the magnitude of the increase in compressive strength and corrosion resistance coefficients.

Using scanning electron microscopy (SEM) and X-ray diffraction (XRD) techniques, it was determined that the internal structure of the uncorroded concrete is dense, with C-S-H gel and AFt distributed uniformly, and the aggregates are tightly bonded, with almost no visible cracks. However, after 90 days of erosion by a 5% Na_2_SO_4_ solution, the concrete structure undergoes significant changes, with a large number of needle-like or rod-shaped AFt forming at the cracks, and their volume increasing. As the Na_2_SO_4_ concentration increases, Ca(OH)_2_ crystals gradually disappear, C-S-H gel becomes more loosely structured, cracks increase in number, and the volume and quantity of AFt further increase, filling the interior of the cracks.

The nanoindentation tests revealed that erosion has a small effect on the modulus of elasticity and hardness of the specimens. However, erosion significantly affected the volume fraction of micropores and calcium hydroxide. The content of micropores gradually increases, while unhydrated cement particles and calcium hydroxide gradually decrease. This indicates an increase in erosion products such as pores and cracks, leading to further deterioration of the concrete.

Based on the mineral phase distribution diagrams of the concrete specimens, it was observed that the modulus of elasticity and hardness showed different contour distributions as the number of micropores increased. Meanwhile, after the specimens were immersed in 8% Na_2_SO_4_ for 90 d, the micropores gradually aggregated to form larger size (tens of micrometers) pores.

## Figures and Tables

**Table 1:** Basic Properties of Cement.

Specific Surface Area	Heat Loss	Admixture	Freezing Time		Bending Strength		Compressive Strength	
			Condensation	Congeal	3d	28d	3d	28d
355 m^2^/kg	2.69%	13.25%	2.18 h	2.68 h	6.2 MPa	8.6 MPa	32.1 MPa	52.8 MPa

**Table 2:** Chemical composition of cement.

SO_3_	SiO_2_	Al_2_O_3_	CaO	Fe_2_O_3_	MgO	Cl^−^
2.33%	20.50%	5.11%	62.23%	5.22%	4.06%	0.03%

**Table 3:** Basic properties of fly ash.

Water Demand	Heat Loss	Water Content	Fineness
94.5	3.83	0.15	10.8

**Table 4:** Mix proportion of Concrete kg/m^3^.

Component	GS1 (W/C = 0.43)	GS2 (W/C = 0.35)	GS3 (W/C = 0.30)
Cement	300	360	425
Water	170	158	150
Fine Aggregate	664	634	647
Coarse Agg.	1155	1167	1173
Fly Ash	100	90	75
Total Mass	2389	2409	2470

Footnotes: W/C: Water-to-cement mass ratio (e.g., GS1: 170 kg water/400 kg cement = 0.43). All values represent absolute mass per cubic meter of concrete (kg/m^3^).

**Table 5:** Surface conditions of concrete specimens immersed in Na_2_SO_4_ solutions of different concentrations.

Concentration	Number	0 d	30 d	60 d	90 d
0%	GS1	** 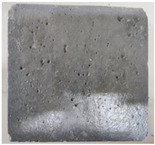 **	** 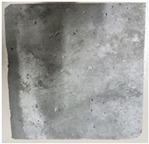 **	** 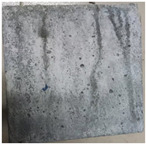 **	** 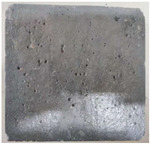 **
	GS2	** 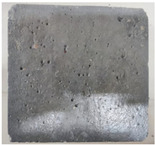 **	** 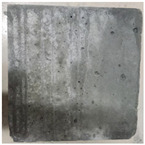 **	** 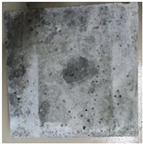 **	** 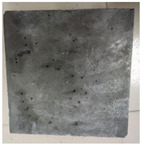 **
	GS3	** 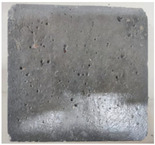 **	** 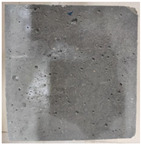 **	** 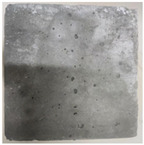 **	** 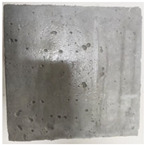 **
5%	GS1	** 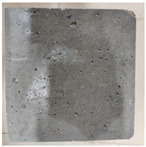 **	** 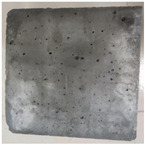 **	** 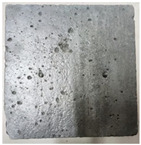 **	** 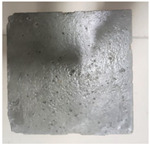 **
	GS2	** 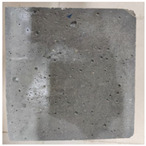 **	** 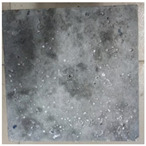 **	** 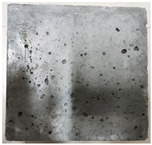 **	** 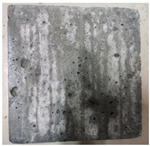 **
	GS3	** 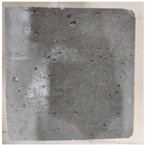 **	** 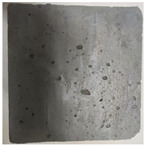 **	** 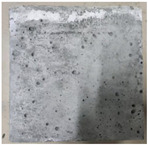 **	** 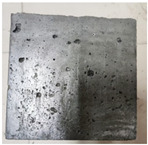 **
8%	GS1	** 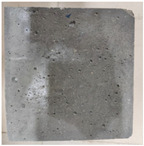 **	** 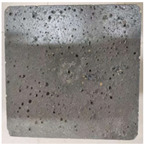 **	** 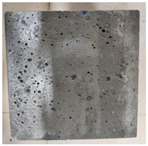 **	** 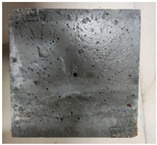 **
	GS2	** 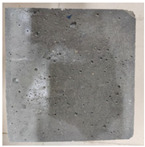 **	** 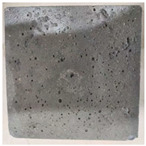 **	** 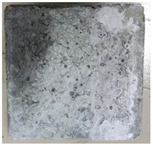 **	** 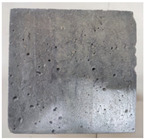 **
	GS3	** 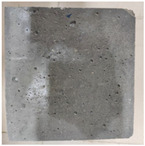 **	** 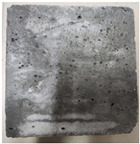 **	** 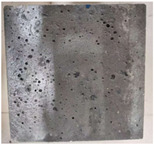 **	** 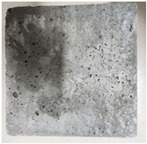 **

**Table 6:** Results of micro-cluster analyses.

Phase	K- Means/GPa	Different Erosion Conditions									
		GS1-0	GS1-W-30	GS1-5-30	GS1-8-30	GS1-W-60	GS1-5-60	GS1-8-60	GS1-W-90	GS1-5-90	GS1-8-90
Micropores	E	11.89 ± 6.00	10.7 ± 6.00	10.16 ± 10.4	10.24 ± 8.60	9.42 ± 6.08	8.43 ± 8.70	7.69 ± 9.40	7.26 ± 8.44	5.64 ± 7.76	5.50 ± 8.40
	H	0.33 ± 0.68	0.39 ± 0.40	0.78 ± 0.70	0.41 ± 0.59	0.21 ± 0.49	0.31 ± 0.69	0.20 ± 0.30	0.31 ± 2.50	0.24 ± 0.36	0.22 ± 0.68
	f	11.11%	12.44%	13.3%	13.78%	14.67%	15.56%	17.78%	17.33%	20%	21.33%
LD C-S-H	E	33.66 ± 6.24	32.24 ± 4.82	33.64 ± 11.8	30.50 ± 9.20	30.74 ± 8.26	29.23 ± 10.40	29.43 ± 5.17	32.69 ± 9.30	26.90 ± 8.60	29.12 ± 4.18
	H	1.69 ± 3.20	1.39 ± 2.31	1.84 ± 0.46	1.51 ± 0.90	1.50 ± 0.16	1.09 ± 0.56	1.43 ± 2.50	2.24 ± 2.55	1.04 ± 1.90	1.16 ± 0.74
	f	32%	33.33%	31.11%	31.56%	32.44%	34.20%	34.22%	35.11%	35.56%	36.45%
HD C-S-H	E	54.13 ± 4.17	42.10 ± 7.20	60.52 ± 19.3	55.03 ± 13.50	47.63 ± 10.87	51.52 ± 10.3	44.51 ± 11.0	52.08 ± 10.50	46.64 ± 8.16	42.55 ± 8.40
	H	2.14 ± 1.50	2.4 ± 1.08	3.49 ± 2.40	1.78 ± 0.52	2.26 ± 0.55	2.91 ± 2.30	2.92 ± 1.58	3.13 ± 2.51	2.78 ± 0.72	2.80 ± 2.30
	f	30.22%	28.44%	30.67%	30.22%	29.78%	28.44%	27.56%	26.67%	25.78%	25.34%
CH/ Fine sand	E	105.02 ± 19.00	86.15 ± 5.65	86.88 ± 18.6	84.38 ± 16.9	82.26 ± 16.54	79.15 ± 8.75	76.24 ± 20.70	73.40 ± 12.20	68.62 ± 20.60	61.14 ± 16.80
	H	5.20 ± 0.96	4.71 ± 0.82	5.75 ± 1.80	3.96 ± 1.87	3.31 ± 1.76	3.80 ± 1.21	3.62 ± 2.52	3.71 ± 2.9	3.04 ± 1.36	3.53 ± 5.10
	f	26.67%	25.33%	24.89%	24.44%	23.11%	21.78%	20.44%	20.89%	18.67%	16.89%

## Data Availability

The original contributions presented in this study are included in the article. Further inquiries can be directed to the corresponding author.
